# Inducing a Concurrent Motor Load Reduces Categorization Precision for Facial Expressions

**DOI:** 10.1037/xhp0000177

**Published:** 2015-11-30

**Authors:** Alberta Ipser, Richard Cook

**Affiliations:** 1 Department of Psychology, City University London

**Keywords:** facial expressions, smile sincerity, mirror neurons, simulation, motor theories

## Abstract

Motor theories of expression perception posit that observers simulate facial expressions within their own motor system, aiding perception and interpretation. Consistent with this view, reports have suggested that blocking facial mimicry induces expression labeling errors and alters patterns of ratings. Crucially, however, it is unclear whether changes in labeling and rating behavior reflect genuine perceptual phenomena (e.g., greater internal noise associated with expression perception or interpretation) or are products of response bias. In an effort to advance this literature, the present study introduces a new psychophysical paradigm for investigating motor contributions to expression perception that overcomes some of the limitations inherent in simple labeling and rating tasks. Observers were asked to judge whether smiles drawn from a morph continuum were sincere or insincere, in the presence or absence of a motor load induced by the concurrent production of vowel sounds. Having confirmed that smile sincerity judgments depend on cues from both eye and mouth regions (Experiment 1), we demonstrated that vowel production reduces the precision with which smiles are categorized (Experiment 2). In Experiment 3, we replicated this effect when observers were required to produce vowels, but not when they passively listened to the same vowel sounds. In Experiments 4 and 5, we found that gender categorizations, equated for difficulty, were unaffected by vowel production, irrespective of the presence of a smiling expression. These findings greatly advance our understanding of motor contributions to expression perception and represent a timely contribution in light of recent high-profile challenges to the existing evidence base.

The discovery of mirror neurons responsive during the execution and passive observation of facial gestures (Ferrari, Gallese, Rizzolatti, & Fogassi, 2003) prompted considerable interest in motor theories of expression perception. Building on models of “action understanding” (Gallese & Sinigaglia, 2011; Kilner, 2011; Schütz-Bosbach & Prinz, 2007; Wilson & Knoblich, 2005), motor theories of expression perception posit that observers simulate expressions within their own motor system—a process likened to covert imitation—thereby aiding perception and interpretation (Goldman & Sripada, 2005; Niedenthal, Mermillod, Maringer, & Hess, 2010; Vitale, Williams, Johnston, & Boccignone, 2014). Various authors have linked aberrant simulation to poor expression recognition in autism spectrum disorder (Dapretto et al., 2006), Möbius syndrome (Bate, Cook, Mole, & Cole, 2013), Parkinson disease (Marneweck, Palermo, & Hammond, 2014), and locked-in syndrome (Pistoia et al., 2010). However, the clinical significance of putative motor contributions to expression perception remains controversial (Bird & Cook, 2013; Hamilton, 2013; Hickok, 2014; Bogart & Matsumoto, 2010).

## Existing Empirical Evidence

Consistent with motor theories of expression perception, previous reports have suggested that blocking facial mimicry impairs performance on expression labeling tasks. For example, observers correctly identified expressions of happiness and disgust less often—their hit rate for these categories was lower on a four-alternative forced-choice (AFC) labeling task—when asked to grip a pen with their teeth, relative to their performance in a free-viewing baseline condition (Oberman, Winkielman, & Ramachandran, 2007). Biting on chopsticks and contracting eyebrow muscles decreased hit rates for happy and angry expressions, respectively, on a 5-AFC labeling task, and both manipulations reduced hit rates for disgusted expressions, relative to observers in a free-viewing control condition (Ponari, Conson, D’Amico, Grossi, & Trojano, 2012). Recipients of botulinum toxin (Botox) injections—a cosmetic procedure resulting in paralysis of muscles in the forehead—underperformed on the Reading the Mind in the Eyes Test (Baron-Cohen, Wheelwright, Hill, Raste, & Plumb, 2001), in which observers have to label affective and communicative expressions from cues present in the eye region, compared with individuals given dermal filler—a cosmetic procedure that does not alter muscle function (Neal & Chartrand, 2011). Female participants (but not males) also took longer to respond during an expression labeling task when asked to clench their teeth, avoid facial movement, and attend to feedback from a skin plaster on their forehead, than when asked to keep their shoulders still (Stel & van Knippenberg, 2008).

Complementary studies have reported motor-induced modulation of expression ratings (Maringer, Krumhuber, Fischer, & Niedenthal, 2011; Rychlowska et al., 2014). Maringer et al. asked participants to rate the “genuineness” of true and false dynamic smiles posed by a computer-generated avatar on a 5-point scale (from *not at all genuine* to *very genuine*). Observers in a blocked mimicry condition were informed that more objective judgments are made when facial movement is kept to a minimum, and were required to hold a pen in their mouths. Observers in a free mimicry condition were given no advice and were free to mimic the expressions. Whereas the ratings given to the true and false smiles by participants in the free mimicry condition differed significantly, the ratings given by participants in the blocked mimicry condition did not (Maringer et al., 2011). In a follow-up study, participants were again asked to rate the genuineness of true and false smiles while wearing a mouth guard, squeezing a ball in their hand, or under free-viewing conditions (Rychlowska et al., 2014). In contrast to the ratings of the two control groups, the ratings given to the true and false smiles by participants in the mouth guard condition did not differ significantly.

## Internal Noise and Bias

Extant studies have described how labeling errors and genuineness ratings vary as a function of motor load. Strikingly, however, no attempt has been made to determine how the precision with which expressions are categorized is affected by a concurrent motor load. Consequently, it is unclear whether performance decrements induced by the foregoing motor manipulations reflect increases in internal noise or are products of bias. Bias errors are made when observers are prone toward a particular percept (perceptual bias) or tend to make or avoid certain responses (response bias). For example, observers judging smile sincerity might incorrectly label a false smile or award it a high genuineness rating, because they tend to *see* smiles as true or because they *choose* to respond “genuine” whenever they experience a degree of indecision. In contrast, precision errors reflect unreliable responses, which fail to vary as a function of smile sincerity. For example, precision errors may arise where internal noise detracts from the description and interpretation of the physical differences between stimuli.

The distinction between performance decrements attributable to bias and internal noise is not trivial. Existing motor theories of expression perception (Goldman & Sripada, 2005; Niedenthal et al., 2010; Vitale et al., 2014) appeal to the idea that the motor system conveys a top-down signal that can be used to disambiguate ambiguous sensory descriptions (e.g., Gilbert & Li, 2013). Manipulations that corrupt or block the top-down signal should therefore induce noisier, but unbiased responding.^1^ Moreover, performance decrements attributable to bias errors can reflect changes in response bias. For example, discomfort experienced by participants asked to grip items between their teeth for prolonged periods may encourage the use of “sad” responses—one of the most frequently encountered emotional labels outside of the lab—and thereby reduce hit rates for happy, disgusted, and angry expressions (Oberman et al., 2007; Ponari et al., 2012). Should motor manipulations modulate labeling or rating performance by altering response strategies, putative motor effects on expression perception may not index genuine *perceptual* effects. Importantly, studies that manipulate motor load between subjects (Maringer et al., 2011; Neal & Chartrand, 2011; Niedenthal, Brauer, Halberstadt, & Innes-Ker, 2001; Ponari et al., 2012; Rychlowska et al., 2014) are particularly vulnerable to differences in response strategy, especially where motor manipulations are confounded with guidance about the effects of mimicry (Maringer et al., 2011; Rychlowska et al., 2014).

## Inconsistent Findings

Not only does the existing evidence base fail to distinguish a loss of categorization precision from bias, but it also contains a great deal of inconsistency. First, several motor manipulations have failed to impair expression labeling. Observers asked to chew gum or grip a pen with their lips showed no sign of diminished expression recognition (Oberman et al., 2007). Nevertheless, (a) both manipulations inhibited overt facial mimicry and disrupted somatosensory feedback and (b) electromyographic recordings revealed that chewing gum induced strong activation of multiple facial muscles (Oberman et al., 2007). Similarly, observers asked to clench their teeth, avoid facial movement, and attend to feedback from a skin plaster on their forehead, exhibited expression labeling accuracy comparable to controls who were simply asked to keep their shoulders still (Stel & van Knippenberg, 2008). Moreover, the application of restrictive gel to the faces of observers *improved* performance in an expression labeling task, relative to a second group who had gel applied to their inner arm (Neal & Chartrand, 2011). Each of these manipulations (chewing gum, gripping a pen with the lips, teeth clenching, avoiding facial movement, attending to feedback from a plaster placed on the forehead, applying restrictive gel to the face) would be expected to interfere with a top-down contribution to expression perception derived from motor simulation.

Second, where observed, motor-induced changes in expression labeling are reported for some expressions, but not for others. For example, in the study reported by (Oberman et al. (2007), gripping a pen between the teeth was associated with reduced hit rates when labeling happy and, to some degree, disgusted expressions, but had no effect on hit rates for fear and sadness. Ponari et al. (2012) found that biting on chopsticks reduced hit rates for disgust, happiness, and fear, but did not significantly alter hit rates for anger, surprise, or sadness. Similarly, eyebrow contraction reduced the hit rate for anger and fear, but labeling of disgust, happiness, and surprise was unaffected. Facial expressions comprise highly correlated changes from across eye and mouth regions (Jack, Garrod, & Schyns, 2014) and are thought to recruit holistic visual processing whereby eye and mouth variation is integrated into a single perceptual representation (Calder, Young, Keane, & Dean, 2000). Manipulations that successfully disrupt movements made with either the upper (e.g., eyebrow contraction) or lower regions (e.g., gripping a pen between teeth) of the face, and thereby block a top-down signal to the visual system, should affect perception of a wide range of facial expressions and gestures. It is therefore unclear why the perception of some expressions, such as sadness (Oberman et al., 2007; Ponari et al., 2012), should be unaffected.

Finally, many individuals with Möbius syndrome, a disorder associated with partial or complete paralysis of the facial muscles, show unimpaired facial expression recognition (Calder, Keane, Cole, Campbell, & Young, 2000; Bogart & Matsumoto, 2010), or evidence of nonspecific visual deficits that extend beyond expression recognition (Bate et al., 2013). Should the motor system make a necessary causal contribution to expression perception—as suggested by reports of motor interference effects in healthy adults—one might expect the expression recognition ability of members of this population to be disproportionately impaired relative to typical observers. Reconciling the findings from Möbius patients, with expression labeling and rating changes induced by motor manipulations in healthy adults, is potentially problematic, a fact highlighted in recent high-profile critiques of motor theories and their evidence base (Caramazza, Anzellotti, Strnad, & Lingnau, 2014; Hickok, 2014).

## The Present Study

In sum, putative motor contributions to expression recognition have stimulated considerable interest, but the findings reported to date are equivocal, and motor theories remain enormously controversial. To advance this literature, the present study introduced a novel technique for investigating motor contributions to expression perception. Using a psychophysical paradigm that overcomes some limitations inherent in simple labeling and rating tasks, we sought to interrogate motor theories of expression perception more rigorously than has been possible to date. Having first confirmed that cues from both the eye and mouth regions contribute to sincerity judgments (Experiment 1), we show that the concurrent production of vowel sounds reduces the precision with which smiles are categorized as sincere or insincere without inducing systematic bias (Experiment 2). We then exclude the possibility that the performance decrement is caused by afferent auditory feedback (Experiment 3), and go on to show that comparable judgments of facial gender are unaffected by the motor load, irrespective of the presence or absence of a smile (Experiments 4 and 5).

## General Method

### Stimuli

**Figure 1 fig1:**
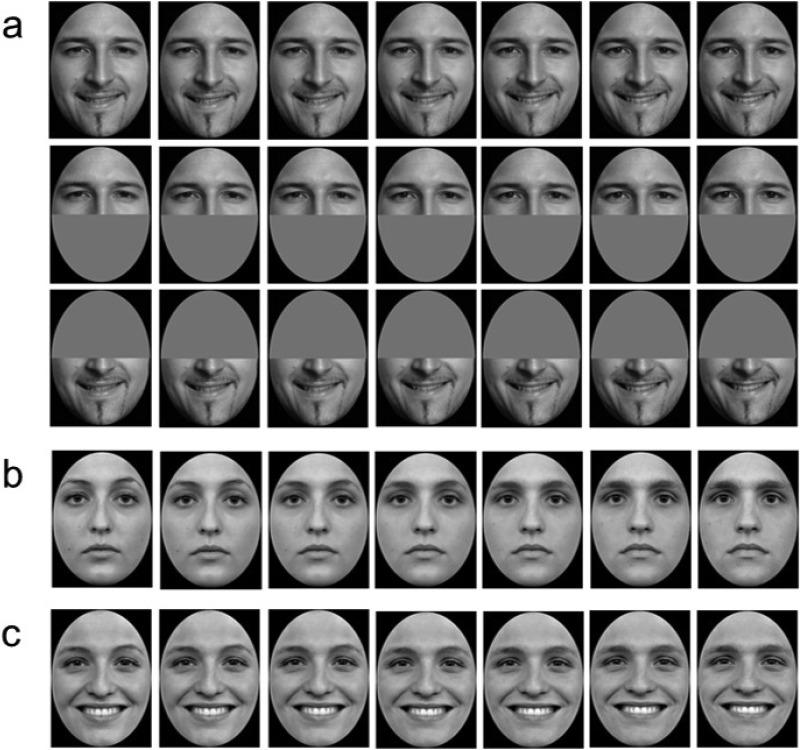
The smile stimuli used in Experiments 1–3, morphing incrementally from 20% sincere (left) to 80% sincere (right) (a). The morph continuum was created using images from the Smile Picture Set (Del Giudice & Colle, 2007). The gender stimuli used in Experiment 4, morphing incrementally from 20% male (left) to 80% male (right) (b). The gender stimuli used in Experiment 5, morphing incrementally from 20% male (left) to 80% male (right) (c). The morph continua from Experiments 4 and 5 were created using images from the Radboud Faces Database (Langner et al., 2010).

Facial stimuli were drawn from morph continua, each comprising seven levels, varying attribute strength from 20% to 80% in increments of 10% (see Figure 1). Image morphing was performed using Morpheus Photo Morpher, Version 3.11 (Morpheus Software, Indianapolis, IN). Stimuli subtended 8° vertically when viewed at a distance of 57.3 cm. The smile morphs used in Experiments 1–3 were created by blending one sincere “enjoyment” smile and one insincere “control” smile from the Smile Picture Set (Del Giudice & Colle, 2007). The set of gender morphs used in Experiments 4 blended one neutral male and one neutral female face taken from the Radboud Faces Database (Langner et al., 2010). The set of gender morphs used in Experiments 5 blended one happy male and one happy female face with the same identity and from the same database as in Experiment 4.[Fig-anchor fig1]

### Procedure

**Figure 2 fig2:**
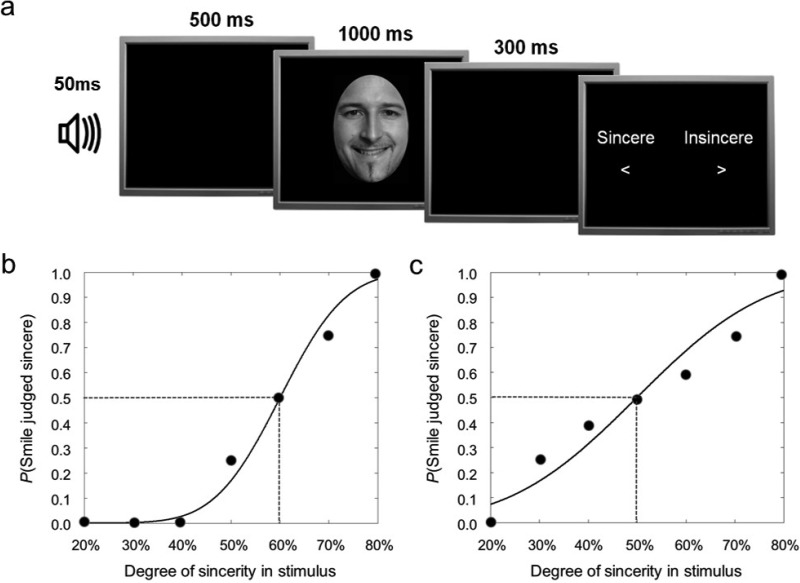
The arrangement of experimental trials (a). In Experiment 1, the tone was task-irrelevant. In Experiments 2–5, observers were required to produce a vowel sound, either /u/ or /i/, cued by the tone. In Experiment 3, observers also passively listened to a vowel (/i/) played through speakers after the tone. Illustration of bias (b). This observer is relatively sensitive to physical changes in the stimulus, but is prone to making “insincere” responses. Illustration of insensitivity (c). This observer exhibits no bias, but the responses vary less with physical changes in stimulus strength.

A common trial format was employed throughout the five experiments described (Figure 2a). Trials began with a 1000-Hz tone of 50-ms duration, followed by an interval of 500 ms, after which a facial stimulus was presented for 1,000 ms. A response screen was presented 300 ms after stimulus offset, prompting participants to make a 2-AFC judgment with their dominant hand. In Experiments 1–3, participants judged whether the facial stimulus depicted a “sincere” or “insincere” smile. In Experiments 4 and 5, participants judged the gender of the face stimulus (“male” or “female”). To make both judgments, observers had to place a given exemplar in a natural category defined by characteristic facial variation; whereas male and female faces differ in facial form and pigmentation (e.g., Cellerino, Borghetti, & Sartucci, 2004), sincere and insincere smiles are defined by characteristic deformations around the eyes and mouth (Del Giudice & Colle, 2007; Niedenthal et al., 2010). The response screen was visible until a response was registered. The next trial began 1,000 ms after the response was made. All experiments were programmed in MATLAB (The MathWorks, Natick, MA) using the Psychophysics Toolbox (Brainard, 1997; Pelli, 1997).[Fig-anchor fig2]

Across all experiments, participants’ responses were modeled by estimating psychometric functions using the Palamedes toolbox (Prins & Kingdom, 2009). Separate cumulative Gaussian functions were fitted for each condition based on 140 observations (20 presentations × 7 stimulus levels) for each participant. Each function estimated two key parameters: The point of subjective equality (PSE) and internal noise. The PSE is a measure of bias that represents the hypothetical stimulus strength equally likely to be judged as sincere or insincere (Experiments 1–3) or male or female (Experiments 4 and 5). Shifts in the PSE can result from changes in response and/or perceptual bias. The noise estimate is a measure of the precision with which stimuli are categorized and was defined as the standard deviation of the symmetric Gaussian distribution underlying each cumulative Gaussian function. Noise estimates are inversely related to the slope of the psychometric function; steep and shallow slopes are associated with low and high noise estimates, respectively. Lower noise estimates indicate that observers can perceive subtle differences in stimulus strength and vary their responses accordingly. Greater noise estimates reveal that participants’ responses are relatively invariant to changes in stimulus strength, indicative of imprecise categorization. Psychometric functions were modeled using a maximum likelihood criterion. PSE and slope measures were free to vary and estimated initially at 50% and 10%, respectively. Guess and lapse rates were fixed at zero. Raw proportions of sincere responses for Experiments 1–3 and male responses for Experiments 4 and 5, used to estimate psychometric functions, are presented as a function of stimulus level in Table 1 and Table 2, respectively.[Table-anchor tbl1][Table-anchor tbl2]

Where psychometric functions were found to vary across conditions, signal detection theory (Green & Swets, 1966) was used to determine whether effects were seen across the stimulus range. For each observer, we estimated that person’s ability to categorize (a) the 20% and 80% levels, (b) the 30% and 70% levels, and (c) 40% and 60% levels. Hits and false alarms were defined according to the categorization task; sincere responses in the presence of the 60%, 70%, and 80% sincere stimuli were treated as hits, and sincere responses in the presence of 20%, 30%, and 40% sincere stimuli as false alarms.^2^ Where participants made no misses/false alarms in a given condition, probabilities of 0.9995 and 0.0005 were assigned for the purposes of the *d*′ calculation.

In each experiment, sample size was determined a priori based on (a) the need to counterbalance the order of three conditions manipulated within subjects and (b) power analysis conducted assuming a large effect size (Cohen, 1988). Ethics clearance was granted by the local ethics committee, and the study was conducted in line with the ethical guidelines laid down in the sixth (2008) Declaration of Helsinki. All participants gave informed consent.

## Experiment 1

It has been suggested manipulations that primarily interfere with upper (e.g., eyebrow contraction) or lower facial mimicry (e.g., biting on a pen) may disproportionately impair perception of expressions characterized by changes in the eye region and mouth region, respectively (Ponari et al., 2012).^3^ It is well established that sincere smiles are associated with contractions of the muscles around the eyes (the orbicularis oculi and the pars lateralis), the so-called Duchenne marker (Duchenne de Boulogne, 1862). Should judgments of smile sincerity be based solely on the Duchenne marker, blocking overt mimicry (or covert simulation) of the mouth through vowel production may induce little perceptual decrement. However, if sincerity judgments also rely on cues present in the mouth region, loading the motor structures associated with mouth movements ought to impair perceptual judgments (Ponari et al., 2012). To aid clear interpretation of our subsequent experiments, we therefore sought to confirm that observers use cues from both the eye and mouth regions when judging the sincerity of the smile morphs (see Figure 1a).

### Method

Twenty-four healthy adults (seven males, *M*_age_ = 31.29 years, one left-handed) participated in Experiment 1. Psychometric functions were estimated for three viewing conditions. In the whole face condition, participants were presented with a smiling face and were free to use cues from the eye region, the mouth region, or both to judge sincerity. In the mouth-only condition, the eye region was occluded, forcing observers to use cues from the mouth region. In the eyes-only condition, the mouth region was occluded, forcing observers to use cues from the eye region. Viewing condition (whole face, eyes-only, mouth-only) was blocked. The order in which participants completed the three blocks was fully counterbalanced. Within each block of 140 trials, the seven levels of sincerity appeared 20 times, in a randomized order.

### Results and Discussion

If observers used cues from both regions when categorizing whole face smiles, performance in the eyes-only and mouth-only conditions should independently predict whole face performance. A multiple regression analysis was therefore conducted in which noise estimates from the whole face condition were regressed onto the noise estimates from the eyes-only and mouth-only conditions. There was no significant correlation between the predictors, *r*(23) = .27, *p* = .196, and the dependent variable was normally distributed, *W*(24) = .96, *p* = .413. Crucially, variability in noise estimates observed in the eyes-only (β = .441, *p* = .009) and in the mouth-only (β = .485, *p* = .005) conditions predicted unique variance in whole face performance. The combined regression model was highly significant, *F*(1, 22) = 12.620, *p* < .001, explaining 54.5% of variability in whole face noise estimates.

Consistent with the existing literature on the Duchenne marker (see Niedenthal et al., 2010, for a review), the results from Experiment 1 indicated that information present in the top half of the face is useful when judging smile sincerity. Critically, however, ability to judge smile sincerity from mouth cues also predicted independent variance in whole face performance, confirming that whole face sincerity judgments depend on cues derived from both the eye and mouth region. Existing simulation accounts of expression perception (Goldman & Sripada, 2005; Niedenthal et al., 2010), therefore, predict that manipulations that load high-level motor areas responsible for planning mouth actions should impair whole face sincerity judgments (Experiments 2 and 3), irrespective of the nature of the top-down contribution; that is, whether it is feature-specific, aiding interpretation of mouth variation only (e.g., Ponari et al., 2012), or global, aiding interpretation of the entire facial configuration (e.g., Friston, 2005; Gregory, 1997).

## Experiment 2

Having determined that cues derived from the mouth area contribute to observers’ judgments of smile sincerity, Experiment 2 examined whether a concurrent motor load would modulate performance on our psychophysical task. To induce motor interference, participants were asked to produce vowel sounds during stimulus presentation. Crucially, motor theories regard covert simulation in high-level motor areas as the core mechanism for action or expression understanding (Gallese & Sinigaglia, 2011; Goldman & Sripada, 2005; Kilner, 2011; Schütz-Bosbach & Prinz, 2007; Vitale et al., 2014; Wilson & Knoblich, 2005). Because vowel production has both planning and production components, it is likely to load high-level motor structures such as the premotor cortex (Rizzolatti & Luppino, 2001).

Manipulations used to block mimicry in previous studies—biting on a pen (Niedenthal et al., 2001; Oberman et al., 2007) or on chopsticks (Ponari et al., 2012), wearing a mouth guard (Rychlowska et al., 2014), 2014), sitting still (Stel & van Knippenberg, 2008), and Botox injections (Neal & Chartrand, 2011)—block the peripheral motor system and may distort, to some degree, afferent feedback where observers make overt movements. However, it is unclear whether the foregoing manipulations effectively engage regions, such as premotor cortex, recruited during the planning and coordination of action (Rizzolatti & Luppino, 2001). Interestingly, a manipulation more likely to load premotor cortex—chewing gum—failed to modulate performance on an expression labeling task (Oberman et al., 2007).

### Method

Twenty-four healthy adults (10 males, *M*_age_ = 28.08 years, two left-handed) participated in Experiment 2. Participants completed the whole face task from Experiment 1 under three conditions. In the baseline condition, participants viewed the stimuli without the requirement to produce a vowel sound. In the remaining conditions, participants produced one of two vowel sounds—either /i/ (pronounced *eeh*, as in *cheese*) or /u/ (pronounced *ooh*, as in *choose*)—cued by the tone at the start of each trial. Participants were required to produce the vowel sound as soon as the tone was detected, and to maintain the sound until the offset of the stimulus image. Auditory responses were recorded and response latencies analyzed using Audacity sound-editing software (http://audacity.sourceforge.net/). The order in which participants completed the conditions was fully counterbalanced across the sample.

### Results and Discussion

**Figure 3 fig3:**
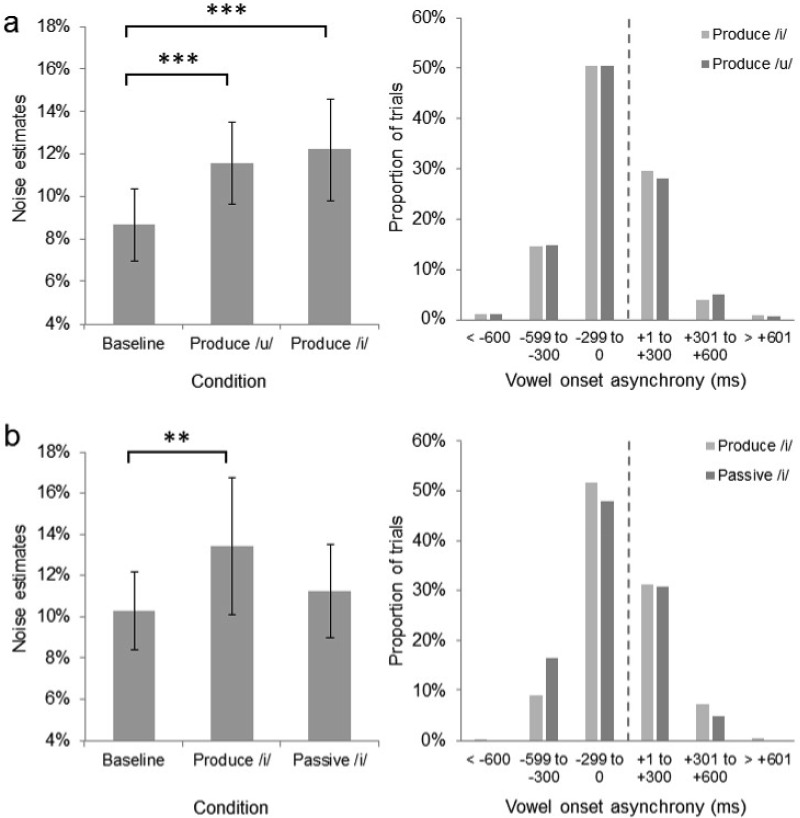
The noise estimates and vowel onset asynchronies from Experiment 2 (a). The noise estimates and vowel onset asynchronies from Experiment 3 (b). Negative asynchronies indicate that vowel production commenced before the onset of the image. Error bars represent 95% confidence intervals. ** *p* < .025. *** *p* < .01.

Analysis of the response latencies indicated that the vowel production task was performed well, with 98.3% of speech sounds produced within ±600 ms of the stimulus onset (see Figure 3a). The noise and PSE estimates were analyzed using analysis of variance (ANOVA) with viewing condition (baseline, produce /i/, produce /u/) as a within-subjects factor. The analyses revealed a significant main effect of viewing condition on noise estimates, *F*(2, 46) = 9.06, *p* = .001, η^2^ = .283. Planned pairwise comparisons revealed significantly lower noise estimates, indicative of greater categorization precision, in the baseline condition (*M* = 8.67, *SD* = 4.02) compared with both the produce /i/ (*M* = 12.20, *SD* = 5.72), *t*(23) = 3.57, *p* = .002, and produce /u/ (*M* = 11.59, *SD* = 4.63) conditions, *t*(23) = 4.16, *p* < .001. The difference between the noise estimates in the produce /i/ and /u/ conditions was not significant, *t*(23) = 0.66, *p* = .518. No significant effect of condition on PSE estimates was found, *F*(2, 46) = 0.40, *p* = .672, η^2^ = .017, indicating that observers’ bias did not differ across the baseline (*M* = 51.17, *SD* = 8.86), produce /u/ (*M* = 50.17, *SD* = 10.14), or produce /i/ (*M* = 50.29, *SD* = 8.62) conditions.[Fig-anchor fig3]

To determine whether the motor load impaired categorization across the entire stimulus range, we estimated each observer’s ability to categorize (a) the 20% and 80% levels, (b) the 30% and 70% levels, and (c) the 40% and 60% levels using signal detection theory (Green & Swets, 1966). Hits and false alarms were defined according to the categorization task; sincere responses in the presence of the 60%, 70%, and 80% sincere stimuli were treated as hits, and sincere responses in the presence of 20%, 30%, and 40% sincere stimuli as false alarms. Where participants made no misses or no false alarms in a given condition, probabilities of 0.9995 and 0.0005 were assigned for the purposes of the *d*′ calculation. The resulting distributions of *d*′ statistics were analyzed using ANOVA with viewing condition (baseline, produce /i/, and produce /u/) and stimulus difference (20–80%, 30–70%, and 40–60%).

The analysis revealed a main effect of viewing condition, *F*(2, 46) = 5.29, *p* = .009, η^2^ = .187. Pairwise comparisons revealed significantly higher attribution sensitivity in the baseline condition (*M* = 4.66, *SD* = 0.96) than in the produce /i/ (*M* = 4.03, *SD* = 1.31), *t*(23) = 2.55, *p* = .018, and produce /u/ (*M* = 4.03, *SD* = 1.16), *t*(23) = 3.32, *p* = .003, conditions. Attribution sensitivity did not differ in the produce /i/ and /u/ conditions, *t*(23) = 0.01, *p* = .992. The analysis also revealed a main effect of stimulus difference, *F*(2, 46) = 126.28, *p* < .001, η^2^ = .846. Attribution sensitivity for the 20% and 80% levels (*M* = 5.45, *SD* = 0.93) exceeded that for the 30% and 70% levels (*M* = 4.61, *SD* = 1.30), *t*(23) = 9.95, *p* < .001, and for the 40% and 60% levels (*M* = 2.66, *SD* = 1.00), *t*(23) = 16.15, *p* < .001. Attribution sensitivity for the 30% and 70% levels also exceeded that of the 40% and 60% levels, *t*(23) = 4.97, *p* < .001. Importantly, however, no Viewing Condition × Stimulus Difference interaction was seen, *F*(4, 92) = 1.54, *p* = .196, η^2^ = .063, suggesting that motor load impaired categorization across the entire stimulus range.

These results indicated that inducing a motor load through vowel production decreased the precision with which smiles were categorized as sincere or insincere, without introducing systematic bias, consistent with motor theories of expression perception (Goldman & Sripada, 2005; Niedenthal et al., 2010; Vitale et al., 2014). In light of the findings from Experiment 1, the motor load may have prevented observers using information around the mouth region to inform their sincerity judgments, thereby reducing categorization precision. Alternatively, the load may have detracted from a top-down contribution in the form of a global interpretation of the facial configuration. The detrimental effect of motor load was not restricted to the highly ambiguous stimuli around the middle of the morph continuum; effects were seen across the range of stimulus intensities.

## Experiment 3

The results of Experiment 2 suggested that concurrent vowel production increases the internal noise associated with expression perception and interpretation, consistent with hypothesized motor contributions to expression perception. It is possible, however, that the decrease in categorization precision did not reflect the presence of the motor load per se, but rather distraction caused by afferent auditory feedback; in other words, the performance decrement may have been induced by the resultant speech sounds, not the speech production itself. Experiment 3 sought to test this alternative account. Participants judged the sincerity of whole face smiles in a baseline viewing condition, a production condition in which participants produced the vowel /i/, and a passive condition in which observers heard the vowel /i/ during stimulus presentation. If the performance decrement observed in Experiment 2 was a product of afferent auditory feedback, the loss of sensitivity should also be induced by the passive auditory signals.

### Method

Twenty-four healthy adults (two males, *M*_age_ = 20.17 years, one left-handed) participated in Experiment 3. The baseline and produce /i/ conditions were identical to those described in Experiment 2. To provide a conservative test of the auditory feedback account, we sought to maximize the salience of the auditory stimuli presented in the passive /i/ condition. During the passive block, each participant encountered 25 tokens of /i/ (five examples of vowels produced by five participants in Experiment 2). Male and female participants heard tokens produced by male and female actors, respectively. The auditory stimulus could occur at 0, ±100, ±200, ±300, ±400, ±500 ms, relative to the onset of the visual stimulus. The distribution of vowel onset asynchronies was yoked to that seen in the production condition in Experiment 2. The offsets of the auditory and visual stimuli in the passive condition were always synchronized. The order in which participants completed the three conditions was fully counterbalanced.

### Results and Discussion

Analysis of the response latencies indicated that the vowel production task in the motor condition was performed well, with 99.1% of speech sounds produced within ±600 ms of the stimulus onset (see Figure 3b). Noise and PSE estimates were analyzed using ANOVA with viewing condition (baseline, produce /i/, and passive /i/) as a within-subjects factor. The analyses revealed a significant main effect of viewing condition on noise estimates, *F*(2, 46) = 3.74, *p* = .031, η^2^ = .140. Pairwise comparisons revealed significantly lower noise estimates, indicative of greater categorization precision, in the baseline condition (*M* = 10.28, *SD* = 4.45) than in the produce /i/ condition (*M* = 13.40, *SD* = 7.81), *t*(23) = 2.51, *p* = .019, replicating the interference effect seen in Experiment 2. Crucially, however, the noise estimates seen in the passive /i/ condition (*M* = 11.23, *SD* = 5.26) did not differ from those seen in the baseline condition, *t*(23) = .96, *p* < .346, arguing against an afferent auditory feedback account of the precision decrement. There was no effect of viewing condition on PSE estimates, *F*(2, 46) = 0.28, *p* = .755, η^2^ = .012, indicating that observers’ bias was comparable in the baseline (*M* = 52.71, *SD* = 6.35), produce /i/ (*M* = 53.08, *SD* = 7.20), and passive /i/ (*M* = 52.21, *SD* = 6.12) conditions.

To determine whether the motor load impaired categorization across the entire stimulus range, we estimated observers’ ability to categorize the 20% and 80% levels, the 30% and 70% levels, and the 40% and 60% levels. The resulting distributions of *d*′ statistics were analyzed using ANOVA with viewing condition (baseline, produce /i/, and passive /i/) and stimulus difference (20–80%, 30–70%, and 40–60%) as within-subjects factors. The analysis revealed a main effect of viewing condition, *F*(2, 46) = 4.36, *p* = .018, η^2^ = .159. Pairwise comparisons revealed significantly lower attribution sensitivity in the produce /i/ condition (*M* = 3.84, *SD* = 1.52) than in the baseline (*M* = 4.46, *SD* = 1.04), *t*(23) = 2.53, *p* = .019, and the passive /i/ (*M* = 4.32, *SD* = 1.18), *t*(23) = 2.08, *p* < .05, conditions. The difference between the baseline and passive /i/ conditions was not significant, *t*(23) = 0.78, *p* = .446. The analysis also revealed a main effect of stimulus difference, *F*(2, 46) = 126.28, *p* < .001, η^2^ = .846. Attribution sensitivity for the 20% and 80% levels (*M* = 5.36, *SD* = 1.27) exceeded that seen for the 70% and 30% levels (*M* = 4.76, *SD* = 1.37), *t*(23) = 2.90, *p* = .008, and for the 60% and 40% levels (*M* = 2.50, *SD* = 1.01), *t*(23) = 17.19, *p* < .001. Attribution sensitivity for the 70% and 30% levels also exceeded that seen for the 60% and 40% levels, *t*(23) = 11.30, *p* < .001. Once again, no Viewing Condition × Stimulus Difference interaction was seen, *F*(4, 92) = 1.54, *p* = .213, η^2^ = .063, suggesting that motor load impaired categorization across the entire stimulus range.

## Experiment 4

The pattern of results observed in Experiments 2 and 3 is consistent with the view that motor processes aid the perception and interpretation of facial expressions via top-down influence (Goldman & Sripada, 2005; Oberman et al., 2007; Vitale et al., 2014). However, these results might also reflect the additional task demands associated with the vowel production task (akin to generic distraction). Afferent somatosensory feedback, or the planning and production components of the concurrent motor task, could load the wider cognitive system, thereby disrupting performance on a wide range of tasks, extending beyond expression perception. Experiment 4 sought to distinguish between these possibilities by testing whether vowel production also modulates categorization precision when observers judge facial gender. Because these tasks have similar demands, a generic distraction account would predict that vowel production should induce comparable precision decrements for gender classification.

### Method

Twenty-four healthy adults (nine males, *M*_age_ = 30.42 years, one left-handed) participated in Experiment 4. Participants were required to judge the gender of whole face stimuli (responding “male” or “female”) drawn from a continuum blending a male and female face (see Figure 1b). Both faces exhibited a “neutral” expression; that is, neither appeared to express emotion. Psychometric functions were modeled under three viewing conditions: baseline, production of /i/, and production of /u/. The order in which participants completed the conditions was again fully counterbalanced. With the exception of the stimuli presented (drawn from a male−female morph) and the judgment made (judging facial gender), the methods were identical to those employed in Experiment 2.

### Results and Discussion

**Figure 4 fig4:**
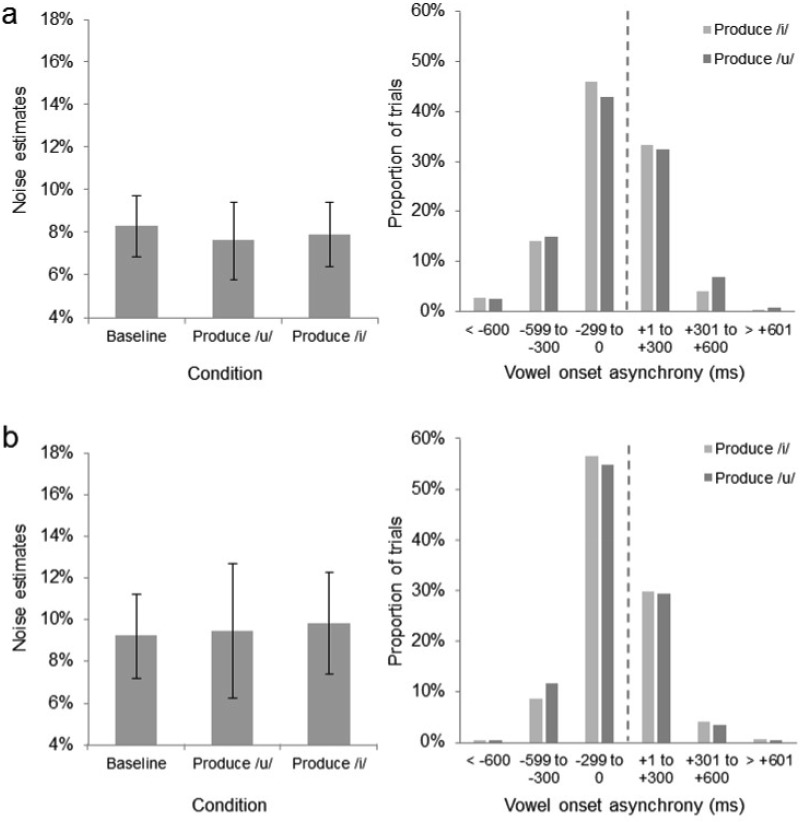
The noise estimates and vowel onset asynchronies from Experiment 4 (a). The noise estimates and vowel onset asynchronies from Experiment 5 (b). Negative asynchronies indicate that vowel production commenced before the onset of the image. Error bars represent 95% confidence intervals.

Analysis of the response latencies indicated that the vowel production task was again performed well, with 97.1% of speech sounds produced within ±600 ms of the onset of the stimulus (see Figure 4a). This level of performance was comparable with that seen in Experiment 2. Noise and PSE estimates were analyzed using ANOVA, with condition (baseline, produce /i/, and produce /u/) as a within-subjects factor. In contrast to Experiment 2, the analysis revealed no effect of condition on noise estimates, *F*(2, 46) = 0.37, *p* = .693, η^2^ = .016, indicating that categorization precision in the baseline (*M* = 8.32%, *SD* = 3.39%), produce /i/ (*M* = 7.66%, *SD* = 4.31%), and produce /u/ (*M* = 7.94%, *SD* = 3.59%) conditions was comparable. Similarly, PSE estimates did not vary across conditions, *F*(2, 46) = 1.96, *p* = .153, η^2^ = .078, indicating that observers’ bias did not differ in the baseline (*M* = 44.23, *SD* = 4.77), produce /i/ (*M* = 44.60, *SD* = 4.36), and produce /u/ (*M* = 46.21, *SD* = 6.31) conditions. The results from Experiment 4 indicated that the precision decrements induced by vowel production in Experiments 2 and 3 did not extend to judgments of facial gender, arguing against a generic distraction account.[Fig-anchor fig4]

## Experiment 5

Experiment 4 indicated that precision decrements induced by vowel production in Experiments 2 and 3 do not simply reflect the increased task demands associated with the concurrent vowel production task. One interpretation of these results is that the categorization of smile sincerity benefits from a top-down contribution from the motor system, one that is not recruited by categorization of facial gender. Consequently, the concurrent motor load impairs sensitivity to smile sincerity, but not facial gender. However, another possibility is that vowel production induces an executive load that is not present in the gender judgment task. It is well known that the sight of actions (e.g., Heyes, 2011) and expressions (e.g., Sato & Yoshikawa, 2007) primes imitative responding. When judging smile sincerity, the requirement to produce vowel sounds is in direct conflict with the tendency to imitate. Because the motor programs to produce a vowel and imitate the smile cannot be discharged simultaneously, an executive process must intervene to resolve the response competition. When judging expression-neutral gender stimuli, however, there is no response competition, and consequently no executive load. The differential effects of vowel production on attribution of smile sincerity and facial gender might reflect the respective presence and absence of this executive load, rather than an expression-specific motor contribution to perception.

Experiment 5 sought to test this executive load account of the effect of vowel production on sincerity judgments. Should the increase in noise seen in Experiments 2 and 3 reflect executive load, a similar decrement should be seen when judging the gender of smiling faces. Importantly, vowel production in the presence of a smiling face induces response competition and consequently an executive load.

### Method

Twenty-four healthy adults (nine males, *M*_age_ = 27.46 years, two left-handed) participated in Experiment 5. Participants were required to judge the gender of whole face facial stimuli drawn from a continuum blending the same male and female faces used in Experiment 4. However, unlike the continuum used in Experiment 4, both of the morphed faces exhibited happy expressions (see Figure 1c). Psychometric functions were again modeled under three viewing conditions: baseline, production of /i/, and production of /u/. The order in which participants completed the conditions was fully counterbalanced. With the exception of the morph stimuli, the methods were identical to those employed in Experiment 4.

### Results and Discussion

Analysis of the response latencies indicated that the vowel production task was again performed well, with 99.1% of speech sounds produced within ±600 ms of the onset of the stimulus (see Figure 4b). This level of performance was comparable to that seen in Experiments 2–4. Noise and PSE estimates were analyzed using ANOVA, with condition as a within-subjects factor. As in Experiment 4, the analysis revealed no effect of condition on noise estimates, *F*(2, 46) = 0.16, *p* = .852, η^2^ = .007, indicating that categorization precision was comparable in the baseline (*M* = 9.23%, *SD* = 4.78%), produce /i/ (*M* = 9.46%, *SD* = 7.58%), and produce /u/ (*M* = 9.82%, *SD* = 5.67%) conditions. PSE estimates were also similar in the baseline (*M* = 46.97, *SD* = 4.47), produce /i/ (*M* = 47.00, *SD* = 5.77), and produce /u/ (*M* = 46.91, *SD* = 8.34) conditions, indicating similar degrees of bias, *F*(2, 46) = 0.002, *p* = .998, η^2^ = .000. The fact that vowel production failed to interfere with attribution of facial gender, irrespective of the presence of a smiling expression, argues against an executive load account of the motor interference effect observed in Experiments 2 and 3.

Finally, we compared noise estimates measured in the baseline conditions across Experiments 2–5. Should the judgments of facial gender be easier than judgments of smile sincerity, the additional task demands (Experiment 4), or the additional executive load (Experiment 5) induced by vowel production, might be sufficient to interfere with sincerity judgments, but insufficient to impair judgments of facial gender. Importantly, however, ANOVA with Experiment (2–5) as a between-subjects factor confirmed that the noise estimates obtained in the baseline conditions did not vary significantly, *F*(3, 92) = 1.01, *p* = .394, η^2^ = .032, confirming that the gender and smile sincerity tasks were of comparable difficulty.

## General Discussion

The present study introduced a novel paradigm for investigating motor contributions to the perception of facial expressions, one that adopts a psychophysical approach, permitting disambiguation of decrements associated with internal noise (e.g., associated with perception and interpretation) from response bias. Having confirmed that observers’ judgments of smile sincerity were based on cues from both the eye and mouth regions (Experiment 1), we went on to show that the presence of a concurrent motor load, induced by vowel production, causes a decrease in the precision with which smiles are categorized; responses varied less as a function of stimulus level (Experiment 2). In Experiment 3, we replicated this effect when observers were required to produce vowels, but not when they passively listened to the same vowel sounds. In Experiments 4 and 5, we found that similar judgments of facial gender were unaffected by vowel production, irrespective of the presence of a smiling expression.

Previous studies have reported that blocking facial mimicry alters expression labeling (Niedenthal et al., 2001; Oberman et al., 2007; Ponari et al., 2012; Stel & van Knippenberg, 2008) and rating performance (Maringer et al., 2011; Rychlowska et al., 2014). Remarkably, however, this is the first study to demonstrate (a) that a concurrent motor task increases the level of internal noise present during categorization of facial expressions, and (b) that reduction in precision does not extend to other types of facial judgment. Crucially, our novel psychophysical paradigm enabled us to exclude the possibility that motor-induced performance decrements reflect changes in response bias. These findings are a timely contribution in light of high-profile challenges to the existing evidence-base for motor theories (Caramazza et al., 2014; Hickok, 2014).

The increase in internal noise induced by vowel production is consistent with simulation accounts of expression perception (Goldman & Sripada, 2005; Niedenthal et al., 2010; Vitale et al., 2014). These models propose that the motor system exerts a top-down influence on perception, useful when interpreting ambiguous expressions. This contribution may be mediated by a reverse simulation process whereby covert mimicry of observed facial expressions aids interpretation by activating motor, somatosensory, and affective states associated with observed expressions (Goldman & Sripada, 2005; Niedenthal et al., 2010). Alternatively, observers may form an initial hypothesis about the emotion conveyed through a visual analysis, which is subsequently tested via covert simulation; for example, the anticipated sensory consequences of the hypothesized state may be compared with feedback from the simulation (see Vitale et al., 2014). Importantly, both reverse simulation and generate-and-test models predict that a concurrent motor load ought to result in reduced precision (e.g., greater internal noise), without introducing a systematic perceptual or decision bias.

An alternative account of the precision decrement seen in Experiments 2 and 3 posits that afferent auditory feedback, and not the motor load itself, induced the loss of sensitivity. Importantly, however, experiencing vowel sounds passively did not interfere with smile categorization (Experiment 3). This finding, together with the fact that predicted sensory consequences of actions are thought to be less salient than unpredicted events triggered exogenously (e.g., Brown, Adams, Parees, Edwards, & Friston, 2013), argues against an afferent auditory feedback account of the perceptual decrement. It is also important to remember that the motor system may contribute to perception through various mechanisms; for example, by modulating processing in low-level visual areas (including V1 and lateral geniculate nucleus; Erisken et al., 2014), by directing the allocation of attention (Bekkering & Neggers, 2002), and by aiding mental rotation (Wexler, Kosslyn, & Berthoz, 1998). Moreover, concurrent vowel production can impair performance through generic distraction (e.g., caused by the additional task demands) and executive load. However, the fact that vowel production did not induce perceptual decrements when judging facial gender (Experiment 4), irrespective of the presence of a smiling expression (Experiments 5), suggests that the interference effect was not a product of low-level sensory modulation, attentional allocation, mental rotation, generic distraction, or executive load.

We speculate that correlated sensorimotor experience may be necessary for the emergence of motor contributions to expression perception (Cook, Bird, Catmur, Press, & Heyes, 2014; Press & Cook, 2015). In the typically developing population, individuals experience a wealth of correlated “seeing” and “doing” during ontogeny; for example, performing a smile or frown frequently predicts the sight of smiling or frowning interactants, respectively. Following this kind of contingent sensorimotor experience, motor programs responsible for expression planning and execution may come to excite predicted visual consequences. Interestingly, perception of arbitrary stimuli, including Gabor patches (Cardoso-Leite, Mamassian, Schütz-Bosbach, & Waszak, 2010) and houses (Kühn, Seurinck, Fias, & Waszak, 2010), can be influenced by motor contributions if paired contingently with action performance during sensorimotor training. In Experiments 4 and 5, vowel production had no influence on judgments of facial gender, suggesting that this perceptual decision does not ordinarily recruit motor processes. However, whether judgments of facial gender have the potential to recruit motor contributions to perception, conceptually similar to those recruited by smile judgments, remains an open empirical question (Press & Cook, 2015).

When healthy adult observers experienced a concurrent motor load, the precision with which they categorized the smile morphs was reduced. This finding is seemingly inconsistent with previous reports that individuals with Möbius syndrome, a congenital condition associated with partial or complete paralysis of the facial muscles, show unimpaired facial expression recognition (Calder, Keane, et al., 2000; Bogart & Matsumoto, 2010), or nonspecific visual deficits (Bate et al., 2013). Drawing conclusions about typical cognitive functioning from atypically developing populations is notoriously difficult (Karmiloff-Smith, 1998). It is possible that this apparent inconsistency reflects the use of insensitive tasks that were unable to detect subtle differences in the expression recognition abilities of Möbius patients. Highly ambiguous judgments, such as categorizing smiles with similar configurations, may be more likely to detect modulations in top-down influence, than tasks requiring the categorization of stereotypical basic emotions (Bate et al., 2013; Calder, Keane, et al., 2000; Bogart & Matsumoto, 2010). Whether the loss of precision observed here extends to other types of expression categorizations remains an open empirical question. Alternatively, expression recognition in individuals with Möbius syndrome may be achieved via a qualitatively different route, drawing on compensatory processes. For example, individuals born with Möbius syndrome, unlike members of the typically developing population, do not experience contingencies between expression production and expression observation, and may therefore develop other sources of top-down modulation.

### Conclusion

The present study reports important new evidence for motor contributions to the perception of facial expressions. Using a novel psychophysical paradigm, we have shown that inducing a concurrent motor load through vowel production causes a loss of perceptual sensitivity, without introducing response bias, when healthy adult observers judge smile sincerity. The perceptual decrement is not a product of afferent auditory feedback and does not extend to similar judgments of facial gender. These findings are consistent with models proposing that the motor system makes a causal contribution to the perception and interpretation of facial expressions (Goldman & Sripada, 2005; Niedenthal et al., 2010; Vitale et al., 2014).

## Figures and Tables

**Table 1 tbl1:** Mean (Standard Deviation) Proportion of Sincere Responses to Each Stimulus Level, in Each Condition, for Experiments 1–3

Experiment	Smile morph level (percent sincere)													
	20%		30%		40%		50%		60%		70%		80%	
Experiment 1														
Whole face	2.7%	(1.2)	2.3%	(0.7)	22.3%	(3.3)	69.4%	(4.2)	90.2%	(2.7)	95.4%	(1.4)	98.1%	(0.7)
Eyes only	4.0%	(1.4)	6.9%	(1.8)	21.5%	(2.9)	61.0%	(5.3)	82.9%	(3.3)	93.8%	(1.6)	95.0%	(1.2)
Mouth only	8.8%	(2.0)	16.7%	(3.1)	38.5%	(4.9)	61.5%	(5.7)	78.8%	(3.9)	88.3%	(3.2)	95.0%	(1.2)
Experiment 2														
Baseline	1.5%	(0.7)	5.8%	(2.6)	20.2%	(4.4)	58.5%	(6.4)	83.8%	(4.1)	95.0%	(2.3)	97.5%	(1.5)
Produce /u/	2.7%	(1.4)	6.7%	(2.0)	22.9%	(5.0)	57.1%	(5.0)	75.8%	(5.0)	91.7%	(2.1)	95.2%	(2.1)
Produce /i/	3.1%	(1.2)	8.8%	(3.4)	22.1%	(4.4)	52.9%	(6.0)	80.0%	(4.7)	90.8%	(2.6)	95.2%	(2.1)
Experiment 3														
Baseline	1.7%	(0.8)	5.6%	(1.8)	24.4%	(4.5)	64.0%	(4.7)	89.0%	(2.3)	96.5%	(1.5)	96.5%	(1.7)
Produce /i/	5.4%	(2.3)	7.3%	(2.8)	26.7%	(4.5)	65.2%	(3.9)	88.3%	(2.3)	92.1%	(2.0)	95.2%	(1.7)
Passive /i/	2.5%	(2.1)	7.1%	(3.7)	23.8%	(5.4)	59.8%	(4.4)	84.2%	(2.7)	96.9%	(1.0)	97.3%	(1.0)

**Table 2 tbl2:** Mean (Standard Deviation) Proportion of Male Responses to Each Stimulus Level, in Each Condition, for Experiments 4 and 5

Experiment	Gender morph level (percent male)													
	20%		30%		40%		50%		60%		70%		80%	
Experiment 4														
Baseline	1.88%	(0.79)	5.00%	(1.53)	27.50%	(3.73)	80.42%	(4.40)	95.21%	(1.69)	98.13%	(0.79)	98.54%	(0.64)
Produce /u/	2.50%	(0.85)	2.71%	(1.13)	26.88%	(3.44)	80.21%	(4.65)	96.25%	(1.25)	98.33%	(0.83)	98.54%	(0.70)
Produce /i/	2.29%	(0.80)	2.50%	(1.09)	21.67%	(3.99)	72.50%	(5.90)	91.25%	(2.95)	97.29%	(1.75)	97.92%	(0.99)
Experiment 5														
Baseline	2.08%	(0.85)	3.75%	(1.10)	20.00%	(3.19)	67.71%	(3.90)	90.83%	(1.99)	97.08%	(1.16)	98.54%	(0.88)
Produce /u/	3.13%	(1.85)	6.25%	(2.76)	21.88%	(4.04)	67.92%	(5.01)	87.08%	(3.90)	95.00%	(1.83)	97.50%	(1.00)
Produce /i/	2.71%	(1.68)	3.75%	(2.00)	18.96%	(3.72)	66.04%	(5.04)	92.50%	(2.15)	96.88%	(1.20)	98.13%	(1.03)

## Footnotes

1 When the concurrent motor task is unrelated to the perceptual judgment (e.g., performing vowel sounds when judging smile sincerity), existing motor theories predict a loss of categorization precision. When concurrent motor tasks are closely related to the judgment (e.g., performing an insincere smile when judging smile sincerity), these models predict perceptual bias.

2 This treatment reflects the nature of the attribution task; that is, participants judged whether stimuli belonged to one of two natural categories (sincere or insincere smiles). This departs from a standard signal detection task whereby a signal is absent on some trials and present in others. In the latter case, it is common to estimate false alarms in the absence of a stimulus and the proportion of hits at varying levels of stimulus intensity.

3 The suggestion that a top-down contribution can selectively modulate perception of the eye or mouth regions is at odds with the finding that expressions are typically defined by correlated eye and mouth variation (Jack et al., 2014), and that expressions are represented holistically (Calder, Young, et al., 2000).
